# Loss of Dna2 fidelity results in decreased Exo1-mediated resection at DNA double-strand breaks

**DOI:** 10.1016/j.jbc.2024.105708

**Published:** 2024-02-02

**Authors:** Aditya Mojumdar, Courtney Granger, Martine Lunke, Jennifer A. Cobb

**Affiliations:** Department of Biochemistry and Microbiology, University of Victoria, Victoria, British Columbia, Canada

**Keywords:** DSB repair pathway choice, Dna2 nuclease, Exo1 nuclease, mutant, 5′ DNA resection, alternative end-joining

## Abstract

A DNA double-strand break (DSB) is one of the most dangerous types of DNA damage that is repaired largely by homologous recombination or nonhomologous end-joining (NHEJ). The interplay of repair factors at the break directs which pathway is used, and a subset of these factors also function in more mutagenic alternative (alt) repair pathways. Resection is a key event in repair pathway choice and extensive resection, which is a hallmark of homologous recombination, and it is mediated by two nucleases, Exo1 and Dna2. We observed differences in resection and repair outcomes in cells harboring nuclease-dead *dna2*-1 compared with *dna2*Δ *pif1*-m2 that could be attributed to the level of Exo1 recovered at DSBs. Cells harboring *dna2*-1 showed reduced Exo1 localization, increased NHEJ, and a greater resection defect compared with cells where *DNA2* was deleted. Both the resection defect and the increased rate of NHEJ in *dna2*-1 mutants were reversed upon deletion of *KU70* or ectopic expression of Exo1. By contrast, when *DNA2* was deleted, Exo1 and Ku70 recovery levels did not change; however, Nej1 increased as did the frequency of alt-end joining/microhomology-mediated end-joining repair. Our findings demonstrate that decreased Exo1 at DSBs contributed to the resection defect in cells expressing inactive Dna2 and highlight the complexity of understanding how functionally redundant factors are regulated *in vivo* to promote genome stability.

Homologous recombination (HR) and nonhomologous end joining (NHEJ) are the canonical pathways of DNA double-strand break (DSB) repair. HR is an error-free pathway requiring extensive 5′ end resection, and NHEJ is an error-prone pathway whereby the ends are joined after minimal processing. DNA resection is the major deciding step between these two pathways (1). However, if resection initiates and HR is not possible, then a more mutagenic alternative (alt) repair pathway can be used as a last resort. Microhomology-mediated end joining (MMEJ) is an alt-end-joining (alt-EJ) pathway that occurs at a high frequency in the absence of yKu70/80 (Ku) or when broken ends are not compatible for direct ligation. MMEJ requires 5′ resection; however, in contrast to HR, the extent of resection in MMEJ is believed to be coordinated with the process of scanning for microhomology in adjacent regions flanking the DSB. The mechanism remains ill defined; however, the repair product from MMEJ contains a deletion corresponding in size to the fragment between the annealed microhomology sequences, which were revealed during resection.

The first responders to a DSB are Ku and Mre11–Rad50–Xrs2 (MRX), and they are important for recruiting additional NHEJ and HR factors (2, 3, 4, 5, 6). The Ku heterodimer also protects the ends from nucleolytic degradation and aids in the localization of Lif1–Dnl4 and Nej1 (3). Dnl4 ligase completes EJ by ligating the DNA with the help of Lif1 and Nej1 (4, 7, 8, 9). The central role of the MRX complex is to tether the loose DNA ends mainly through the structural features of Rad50 (10, 11) and to initiate resection through the nuclease activity of Mre11 (12). Sae2 interacts with the MRX complex and activates Mre11 nuclease activity, which forces Ku dissociation. Ku disengagement at the DSB coincides with the initiation of 5′ to 3′ end resection by two long-range resection nucleases, Dna2 in complex with Sgs1 helicase, and Exo1 (12, 13, 14). Dna2 and Exo1 nucleases show functional redundancy as Exo1 drives long-range resection in the absence of Dna2 and vice versa (14). The interplay between repair factors in the two canonical pathways regulates the initiation of resection in part through antagonistic relationships between Ku and Exo1 and between Nej1 and Dna2, wherein Nej1 inhibits interactions of Dna2 with Sgs1 and with Mre11 and Sae2 (5, 6, 15, 16).

Dna2 is mutated in a myriad of human cancers, but because of its essential role in processing Okazaki fragments and other intermediates at replication forks, *DNA2* cannot be deleted (17, 18, 19, 20, 21). However, in yeast *dna2*Δ, lethality can be suppressed by mutation of *PIF1*, a gene encoding a DNA helicase that does not function in 5′ resection at DSBs (22). In the absence of Mre11 nuclease activity, resection initiates primarily through Dna2, not through Exo1 (23, 24, 25). However, there is a gap in understanding the regulation of Dna2 at DSBs as nuclease-deficient *dna2*-1 (P504S) shows greater sensitivity to DSB-inducing agents compared with *dna2*Δ *pif1*-m2 (Fig. 1*A*) (26, 27).

**Figure 1:**
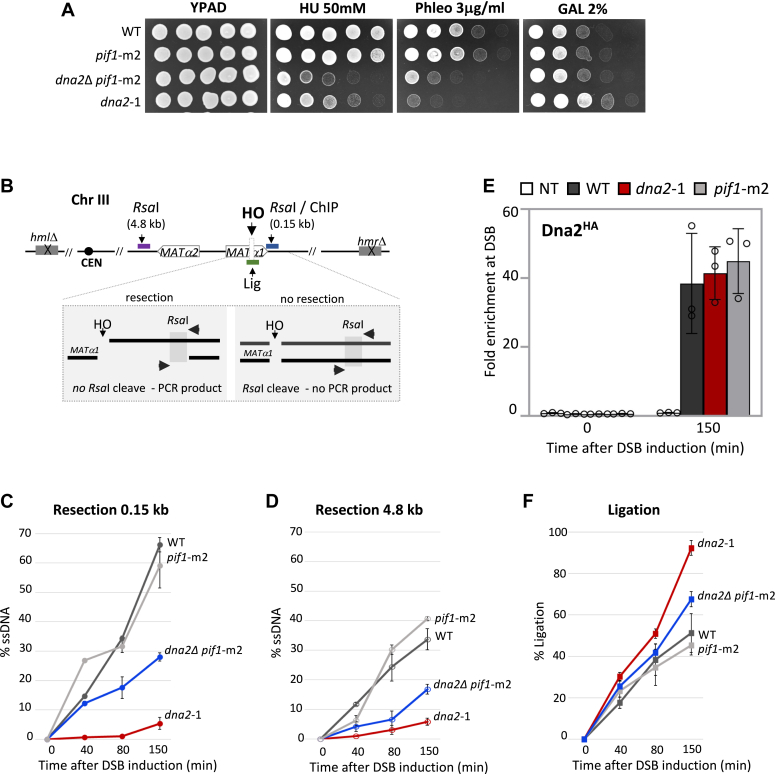
**Nuclease-deficient *dna2*-1 leads to compromised resection at DSB.***A*, fivefold serial dilutions of the strains—WT (JC-727), *dna2*Δ *pif1*-m2 (JC-6005), *pif1*-m2 (JC-6006), and *dna2*-1 (JC-6007) were spotted on YPAD, 50 mM HU, 3.0 μg/ml phleomycin, and 2% galactose containing plates. *B*, schematic representation of regions around the HO cut site on chromosome III. The ChIP primers used in this study correspond to 0.15 kb (*blue*) from the DSB, and the end-joining primers flank the HO cut site (Lig, *green*). The qPCR resection assay relies on two RsaI sites located 0.15 kb (*blue*) and 4.8 kb (*purple*) from the DSB. *C* and *D*, qPCR-based resection assay of DNA 0.15 kb and 4.8 kb away from the HO DSB, as measured by % ssDNA, at 0, 40, 80, and 150 min post DSB induction in cycling cells in WT (JC-727), *dna2*Δ *pif1*-m2 (JC-6005), *pif1*-m2 (JC-6006), and *dna2*-1 (JC-6007). *E*, enrichment of Dna2^HA^ at 0.15 kb from DSB 0 min (no DSB induction) and 150 min after DSB induction in WT (JC-4117), *dna2*-1 (JC-5707), *pif1*-m2 (JC-6130), and no tag control (JC-727) was determined. The fold enrichment is normalized to recovery at the *PRE1* locus. *F*, qPCR-based ligation assay of DNA at HO DSB, as measured by % Ligation, at 0, 40, 80, and 150 min in cycling cells in glucose post DSB. Strains used were WT (JC-727), *dna2*Δ *pif1*-m2 (JC-6005), *pif1*-m2 (JC-6006), and *dna2*-1 (JC-6007). ChIP, chromatin immunoprecipitation; DSB, double-strand break; qPCR, quantitative PCR.

Here, we determined that the dominant negative effects of *dna2*-1 were caused by decreased localization of Exo1, a nuclease functionally redundant with Dna2 in DSB repair. In *dna2*-1 mutant cells, Ku-dependent NHEJ increased and Exo1-dependent 5′ resection decreased. By contrast, in dna2Δ *pif1*-m2 mutants, Ku70 and Exo1 recruitment to the break remained indistinguishable from WT, but EJ repair occurred mainly through MMEJ. These results demonstrate that Exo1 recovery is impacted by the physical presence of Dna2 and that the interplay between these two nucleases regulates key events that drive repair pathway choice, including the ratio of NHEJ and MMEJ.

## Results

### Nuclease-deficient dna2-1 shows abrogated resection at DSB

Cells harboring nuclease-dead *dna2*-1 were more sensitive than *dna2Δ pif1*-m2 mutants to phleomycin, an agent that causes DNA DSBs, but less sensitive to hydroxyurea, an agent inducing replication stress (Fig. 1*A*, (26, 27)). While sensitivities to various genotoxic stressors have been previously reported with *dna2* mutants, there has been little work explaining why *dna2*-1 mutants show greater sensitivity to DSB-causing agents compared with cells harboring the deletion of *DNA2* or its binding partner, *SGS1* (Figs. 1*A* and S1). This prompted our side-by-side investigation of *dna2Δ pif1*-m2 and *dna2*-1 in DSB repair. Our aims were to evaluate DNA resection, a key early step in HR and then to determine the impact of the *dna2* mutations on the functionality of the other DSB repair factors.

Dna2 functions in long-range resection and can compensate for Mre11 to initiate resection (14). To this end, resection was determined at two locations, 0.15 and 4.8 kb from the HO-induced DSB using a quantitative PCR (qPCR)–based approach that relies on *Rsa*I as previously described (16, 28). Resection produces ssDNA, and if resection goes beyond the *Rsa*I recognition sequence, then the site is not cleaved and can be amplified by PCR (Fig. 1*B*, loci in *blue* and *purple*). Resection at the time points (0–150 min) was similar at both distances from the break in *pif1*-m2 and WT, indicating that the loss of *PIF1* activity did not impact DNA processing at DSB (Fig. 1, *C* and *D*). Furthermore, when we performed chromatin immunoprecipitation (ChIP) at the HO-induced DSB, Dna2^HA^ levels in *pif1*-m2 mutants were indistinguishable from WT (Fig. 1*E*), reinforcing earlier work showing that the disruption of *PIF1* did not impact DSB repair (22). Upon deletion of *DNA2*, resection decreased by approximately twofold, with a slightly greater defect at the distance 4.8 kb from the break (Fig. 1, *C* and *D*). A more pronounced defect was observed in *dna2*-1 mutants as resection was abrogated at both distances (Fig. 1, *C* and *D*). Dna2 recovery in *dna2*-1 mutants was unaltered (Fig. 1*E*), suggesting that the physical association of this nuclease-dead mutant at the break had a dominant negative impact.

We also observed that *dna2*-1 mutant cells survived better than *dna2Δ pif1*-m2 and WT on 2% GAL (Fig. 1*A*). The genetic background of these cells includes *hml*Δ *hmr*Δ, which prevents HR. Survival on galactose therefore correlates with mutagenic EJ repair, which prevents subsequent HO-cutting as opposed to survival on phleomycin, which creates multiple DSBs throughout the genome that can repair by HR.

To complement the survival assays, we performed an EJ ligation experiment where the DSB was induced with galactose for 2 h before cells were washed and released into glucose to prevent further recutting. At the indicated time points, genomic DNA was prepared, and qPCR was performed with primers spanning the HO recognition site as previously described (Fig. 1*B*, locus in *green*; 9). The rate of EJ increased more in *dna2*-1 compared with *dna2Δ pif1*-m2 mutants (Fig. 1*F*). Increased EJ might arise naturally because of decreased HR but could also arise from more NHEJ factors at the DSB in *dna2*-1 mutants.

### NHEJ factors at DSBs in dna2 mutants

**Figure 2:**
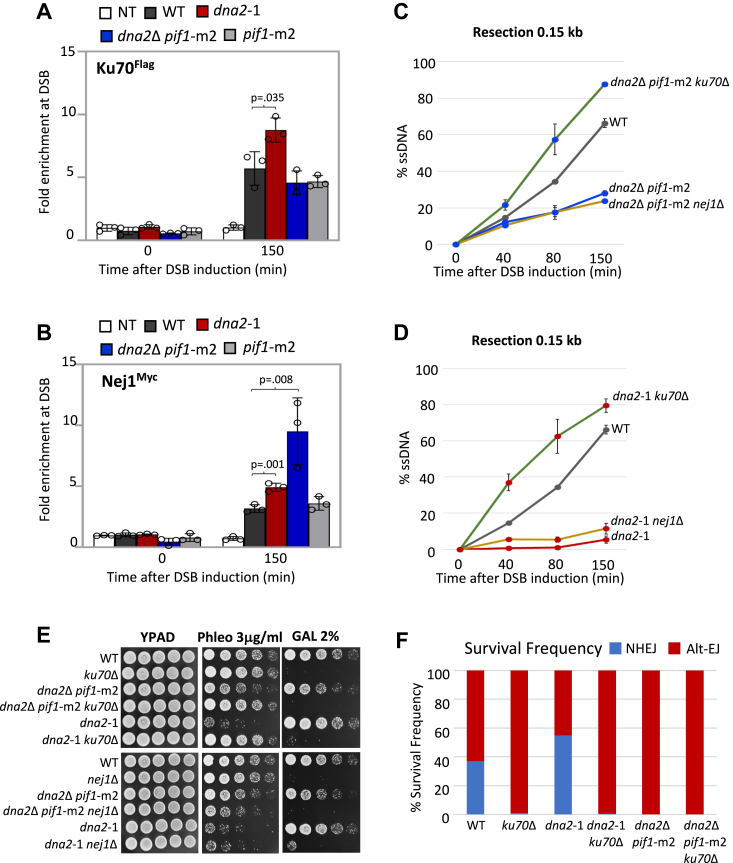
**Nuclease-deficient *dna2*-1 promotes end joining at DSB.***A*, enrichment of Ku70^Flag^ at 0.15 kb from DSB, 0 min (no DSB induction) and 150 min after DSB induction in WT (JC-3964), *dna2*-1 (JC-6237), *dna2*Δ *pif1*-m2 (JC-6068), *pif1*-m2 (JC-6069), and no tag control (JC-727) was determined. The fold enrichment is normalized to recovery at the *PRE1* locus. *B*, enrichment of Nej1^Myc^ at 0.15 kb from DSB, 0 min (no DSB induction) and 150 min after DSB induction in WT (JC-1687), *dna2*-1 (JC-5479), *dna2*Δ *pif1*-m2 (JC-6099), *pif1*-m2 (JC-6132), and no tag control (JC-727). *C* and *D*, qPCR-based resection assay of DNA 0.15 kb away from the HO-DSB, as measured by % ssDNA, at 0, 40, 80, and 150 min post DSB induction in cycling cells in WT (JC-727), *dna2*Δ *pif1*-m2 (JC-6005), *ku70*Δ *dna2*Δ *pif1*-m2 (JC-6128), *nej1*Δ *dna2*Δ *pif1*-m2 (JC-6060), *dna2*-1 (JC-6007), *ku70*Δ *dna2*-1 (JC-5942), and *nej1*Δ *dna2*-1 (JC-5670). *E*, fivefold serial dilutions of the strains used in (*C*) and (*D*) were spotted on YPAD, 3.0 μg/ml phleomycin, and 2% galactose containing plates. *F*, survival frequencies depicting the ratio of NHEJ (*blue*) and alt-EJ–MMEJ (*red*) repair frequencies in WT (JC-5903), *ku70*Δ (JC-6195), *dna2*-1 (JC-6105), *ku70*Δ *dna2*-1 (JC-6273), *dna2*Δ *pif1*-m2 (JC-6181), and *ku70*Δ *dna2*Δ *pif1*-m2 (JC-6280). For all ChIP experiments, the error bars represent the SD of three experimental replicates. Significance was determined using a one-tailed, unpaired Student’s *t* test. The *p* value of significant differences compared with WT is shown in the figure. alt-EJ, alt-end joining; ChIP, chromatin immunoprecipitation; DSB, double-strand break; MMEJ, microhomology-mediated end joining; NHEJ, nonhomologous end-joining.

Prior to comparing the impact of the *dna2* mutants on factors driving resection, we determined the localization of proteins essential for NHEJ. Ku70 recovery at DSB increased in *dna2*-1 mutant cells but not in *dna2*Δ *pif1*-m2 mutants (Fig. 2*A*). By contrast, the recovery of Nej1 increased significantly in both mutants, with *dna2*Δ *pif1*-m2 showing a greater increase (Fig. 2*B*). These data highlight the antagonistic relationship between Dna2 and Nej1 at DSBs (5). The recovery of the other canonical NHEJ factors, Lif1 and Dnl4, in cells harboring either of the *dna2* mutant, was indistinguishable from WT (Fig. S2, *A* and *B*).

We wanted to determine whether preventing NHEJ would reverse the resection defect of the *dna2* mutants and potentially the dominant negative effect of *dna2*-1 in HR repair. In addition to their essential function in NHEJ, both Ku70 and Nej1 inhibit resection (Fig. S2*E*). In alignment with our earlier work, deletion of *KU70*, but not the other core NHEJ factors, reversed the resection defect in both *dna2* mutants (Figs. 2, *C* and *D* and S2, *C* and *D*; (9)). These data indicate that alleviation of the resection defects by *ku70*Δ was independent of *PIF1* status, which differs between the two *dna2* mutants. Correlating with the rescue in resection, deletion of *KU70*, but not *NEJ1*, suppressed the phleomycin sensitivities of both *dna2*Δ *pif1*-m2 and *dna2*-1 mutants (Fig. 2*E*). Altogether, suppressing the dominant negative effect of *dna2*-1 was specific to the loss of Ku70 rather than disruption of NHEJ by *ku70*Δ as HR-mediated repair was restored in *dna2*-1 *ku70*Δ mutants but not in *dna2*-1 *nej1*Δ mutants.

To determine the type of EJ that can proceed in these mutants, we utilized a reporter system containing a *URA3* marker flanked by two inverted HO recognition sites (Fig. S2*F*) (29). If both sites are cleaved simultaneously, noncompatible ends are generated and alt-EJ–MMEJ is used. However, because cutting at both sites is not perfectly coordinated, each single cut can still be repaired by NHEJ as previously described (29). In WT cells, the relative frequency of NHEJ and alt-EJ–MMEJ as determined by growth on -URA was 37% and 63%, respectively (Fig. 2*F*). Notably, the relative frequencies of NHEJ and MMEJ differed between the two *dna2* mutants. The frequency of NHEJ increased to 55% in *dna2*-1 mutants (Fig. 2*F*). By contrast, when *DNA2* was deleted, alt-EJ–MMEJ was the preferred EJ pathway, which we found surprising given Ku70 was similarly recovered at the break site in *dna2*Δ *pif1*-m2 and WT cells (Fig. 2, *A* and *F*). Consistent with previous work, upon *KU70* deletion, EJ occurred through alt-EJ–MMEJ, and the increased NHEJ seen in *dna2*-1 mutants was reversed by *ku70*Δ (Fig. 2*F*).

### Nuclease-deficient dna2-1 suppresses Exo1 recruitment to DSB

**Figure 3:**
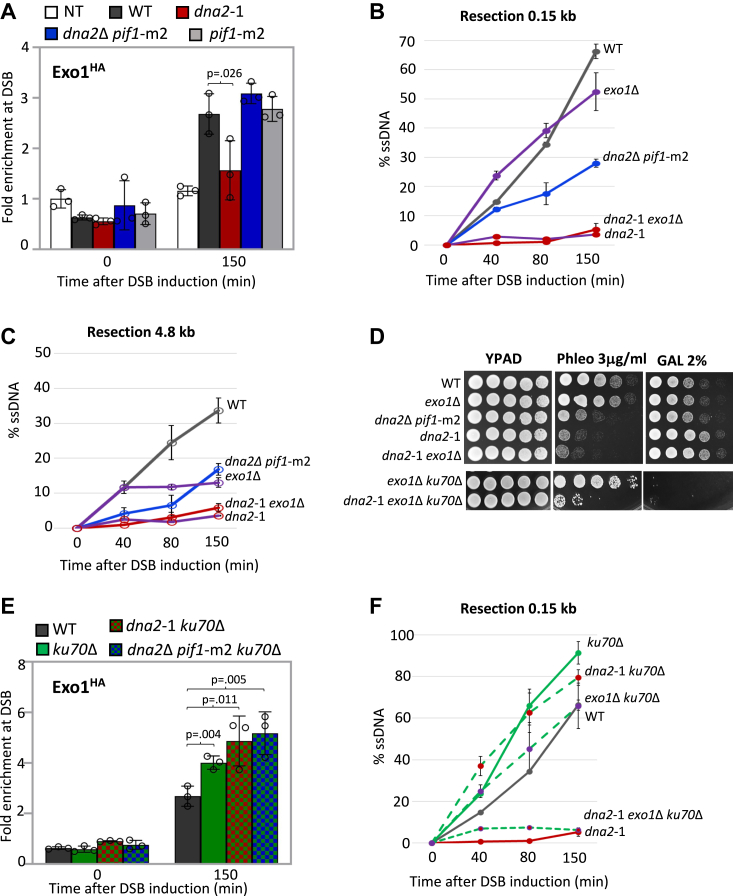
**Nuclease-deficient *dna2*-1 suppresses Exo1 recruitment at DSB.***A*, enrichment of Exo1^HA^ at 0.15 kb from DSB 0 min (no DSB induction) and 150 min after DSB induction in WT (JC-4869), *dna2*-1 (JC-6020), *dna2*Δ *pif1*-m2 (JC-6115), *pif1*-m2 (JC-6117), and no tag control (JC-727) was determined. The fold enrichment is normalized to recovery at the *PRE1* locus. *B* and *C*, qPCR-based resection assay of DNA 0.15 kb and 4.8 kb away from the HO DSB, as measured by % ssDNA, at 0, 40, 80, and 150 min post DSB induction in cycling cells in WT (JC-727), *exo1*Δ (JC-3767), *dna2*-1 (JC-6007), *exo1*Δ *dna2*-1 (JC-5692), and *dna2*Δ *pif1*-m2 (JC-6005). *D*, fivefold serial dilutions of WT (JC-727), *exo1*Δ (JC-3767), *dna2*-1 (JC-6007), *exo1*Δ *dna2*-1 (JC-5692), *dna2*Δ *pif1*-m2 (JC-6005), *ku70*Δ *exo1*Δ (JC-3837), and *ku70*Δ *exo1*Δ *dna2*-1 (JC-6025) were spotted on YPAD, 3.0 μg/ml phleomycin, and 2% galactose containing plates. *E*, enrichment of Exo1^HA^ at 0.15 kb from DSB, 0 min (no DSB induction) and 150 min after DSB induction in WT (JC-4869), *ku70*Δ (JC-6018), *ku70*Δ *dna2*-1 (JC-6215), and *ku70*Δ *dna2*Δ *pif1*-m2 (JC-6213) was determined. The fold enrichment is normalized to recovery at the *PRE1* locus. *F*, qPCR-based resection assay of DNA 0.15 kb away from the HO DSB, as measured by % ssDNA, at 0, 40, 80, and 150 min post DSB induction in cycling cells in WT (JC-727), *ku70*Δ (JC-1904), *dna2*-1 (JC-6007), *ku70*Δ *dna2*-1 (JC-5942), *ku70*Δ *exo1*Δ (JC-3837), and *ku70*Δ *exo1*Δ *dna2*-1 (JC-6025). For all ChIP experiments, the error bars represent the SD of three experimental replicates. Significance was determined using a one-tailed unpaired Student’s *t* test. The *p* value of significant differences compared with WT is shown in the figure. ChIP, chromatin immunoprecipitation; DSB, double-strand break; qPCR, quantitative PCR.

To bring further insight to events underlying the *dna2*-1 phenotype, we next determined the impact of *dna2*-1 and *DNA2* deletion on the localization of other factors important for resection, namely Exo1 nuclease and the nuclease-associated factors, Sae2 and Sgs1. The recovery of Sgs1 and Sae2 was not altered in either mutant background (Fig. S3, *A* and *B*). By contrast, Exo1 was significantly reduced in *dna2*-1 to almost the level of the nontagged control, whereas its recovery in *dna2*Δ *pif1*-m2 was like WT (Fig. 3*A*). These data indicate that Exo1 localization was inhibited by the presence of nuclease-deficient Dna2 at the break rather than the intrinsic loss of Dna2 nuclease activity.

We next compared resection and survival when the *dna2* mutants were combined with deletion of *EXO1*. In line with previous observations, short-range resection (0.15 kb) decreased modestly in *exo1*Δ single mutant cells (Fig. 3*B*), but there was an approximately twofold decrease in long-range resection (4.8 kb) 80 to 150 min after DSB induction (Fig. 3*C*) (14, 16). We could not determine resection when both nucleases were deleted because of synthetic lethality (SL) (14). However, resection at both distances from the break and phleomycin sensitivity in *dna2*-1 *exo1*Δ mutants was indistinguishable from *dna2*-1 (Fig. 3, *B*–*D*). One explanation for the marked decrease in resection in *dna2*-1 was that expression of this mutant directly blocked the association of Exo1 with DSBs. However, Exo1 was similarly recovered in WT cells, which are *DNA2*+ *PIF1*+ and *dna2*Δ *pif1*-m2 mutant cells, suggesting neither *PIF1* status nor the presence of Dna2 *per se* directly affected Exo1 recovery. A more plausible model, based on previous work showing Exo1 to be negatively regulated by the presence of Ku (15), is that the pronounced resection defect in *dna2*-1 mutants resulted from decreased Exo1 because of increased Ku, in addition to the loss of Dna2 nuclease activity.

Indeed, upon deletion of *KU70*, Exo1 recovery increased at the DSB in *dna2*-1 mutants (Fig. 3*E*). These data were also consistent with suppression of *dna2*-1 phleomycin sensitivity by *KU70* deletion being Exo1 dependent (Figs. 2*D* and 3*D*). Resection remained low in *dna2*-1 *exo1*Δ *ku70*Δ triple mutants, as did growth on phleomycin and 2% GAL because both main DSB repair pathways, HR and NHEJ, were disrupted (Fig. 3, *D* and *F*). However, alt-EJ–MMEJ was still functional, which could provide some insight as to how a small percentage of triple mutants survived on phleomycin (Figs. 3*D* and S3*C*). Finally, Exo1 recovery also increased when *KU70* was deleted in *dna2*Δ *pif1*-m2 (Fig. 3*E*). These data help explain why the resection defect in *dna2*Δ *pif1*-m2 mutants was suppressed by *ku70*Δ but not by *nej1*Δ (Fig. 2*C*), as our earlier work showed the inhibitory effect of Nej1 to be unrelated to Exo1 activity (5, 6, 9).

### Overexpression of Exo1 restores the resection defect in dna2-1 cells

**Figure 4:**
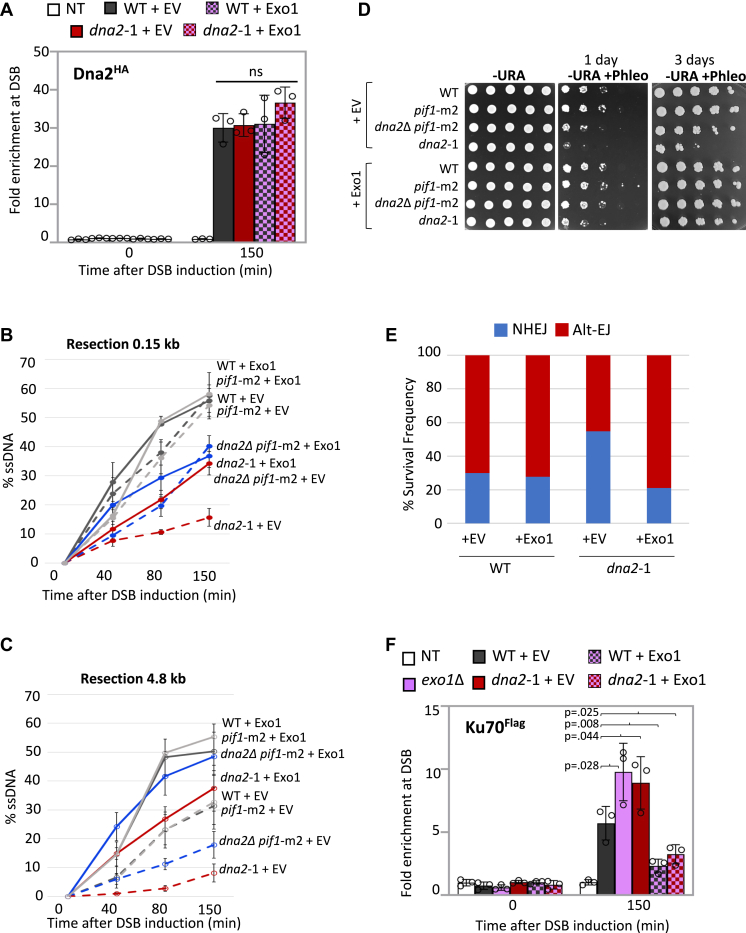
**Overexpression of Exo1 restores the resection defect in *dna2*-1 cells.***A*, enrichment of Dna2^HA^ at 0.15 kb from DSB, 0 min (no DSB induction) and 150 min after DSB induction in WT (JC-4117), *dna2*-1 (JC-5707) with 2-micron empty vector (pRS426) or 2-micron plasmid encoding Exo1 (pEM-EXO1), and no tag control (JC-727). The fold enrichment is normalized to recovery at the *PRE1* locus. *B* and *C*, qPCR-based resection assay of DNA 0.15 kb and 4.8 kb away from the HO DSB, as measured by % ssDNA, at 0, 40, 80, and 150 min post DSB induction in cycling cells in WT (JC-727), *dna2*Δ *pif1*-m2 (JC-6005), and *dna2*-1 (JC-6007) with either 2-micron empty vector (pRS426) or 2-micron plasmid encoding Exo1 (pEM-EXO1). *D*, fivefold serial dilutions of WT (JC-727), *dna2*Δ *pif1*-m2 (JC-6005), *pif1*-m2 (JC-6006), and *dna2*-1 (JC-6007) with + pEM-EXO1 or empty vector. Cells were spotted on -URA plate ± 3.0 μg/ml phleomycin and allowed to grow for 1 to 3 days. *E*, frequency ratio of NHEJ (*blue*) and alt-EJ–MMEJ (*red*) in WT (JC-5903) and *dna2*-1 (JC-6105) with either the 2-micron plasmid encoding Exo1 (pRS425-EXO1) or the empty vector (pRS425) ctrl. *F*, enrichment of Ku70^Flag^ at 0.1 5kb from DSB, 0 min (no DSB induction) and 150 min after DSB induction in WT (JC-3964), *dna2*-1 (JC-6237) with either 2-micron empty vector (pRS426) or 2-micron plasmid encoding Exo1 (pEM-EXO1), *exo1*Δ (JC-6242), and no tag control (JC-727) was determined. The fold enrichment is normalized to recovery at the *PRE1* locus. For all ChIP experiments, the error bars represent the SD of three experimental replicates. Significance was determined using a one-tailed unpaired Student’s *t* test. The *p* value of significant differences compared with WT is shown in the figure. alt-EJ, alt-end joining; ChIP, chromatin immunoprecipitation; DSB, double-strand break; NHEJ, nonhomologous end-joining; qPCR, quantitative PCR.

Our data thus far support a model whereby the dominant negative effect of *dna2*-1 on resection stemmed from increased Ku70 at the DSB, which in turn inhibited Exo1 localization. This was supported by genetic analysis where deletion of *KU70* in *dna2*-1 resulted in increased Exo1 recovery, increased resection, and decreased sensitivity to phleomycin. We next wanted to determine whether increasing the level of Exo1 could rescue the dominant negative effect of *dna2*-1 mutants. We utilized a 2μ URA3 plasmid encoding Exo1 (pEM-EXO1) that was previously engineered to investigate the inhibition of Exo1 by Ku (15). Of note, expression of Exo1 did not alter Dna2 recovery in WT or *dna2*-1 mutants (Fig. 4*A*). Resection at 0.15 kb in *dna2*-1 + pEM-EXO1 increased to the level seen in *dna2*Δ *pif1*-m2 mutants, although resection in both remained lower than WT (Fig. 4*B*). At the further distance, 4.8 kb from the DSB, Exo1 expression in both *dna2* mutants resulted in a greater rescue where resection in *dna2*-1 + pEM-EXO1 was like WT + empty vector, and resection in both *dna2*Δ *pif1*-m2 and WT + pEM-EXO1 was similarly increased (Fig. 4*C*). Highlighting the link between resection and *in vivo* DSB repair, phleomycin sensitivity decreased in both *dna2* mutants expressing Exo1 most notably in *dna2*-1 mutants after 3 days of growth (Fig. 4*D*). Resection and phleomycin sensitivity in *pif1*-m2 + pEM-EXO1 was indistinguishable from WT (Figs. 4*D* and S4, *A* and *B*).

Finally, we wanted to determine whether ectopic expression of Exo1 in *dna2*-1 mutants could restore the balance of NHEJ and alt-EJ–MMEJ repair. Again, we utilized the reporter system where NHEJ and alt-EJ-MMEJ were distinguished by growth on -URA media and where cells were transformed with either pRS425-EXO1 for ectopic expression of Exo1 from a 2μ LEU2 plasmid or empty vector (Fig. S2*F*). Increased NHEJ and the relative frequency of EJ in *dna2*-1 mutants was reversed upon Exo1 expression (Figs. 2*F* and 4*E*). These findings are consistent with short-range resection and decreased Ku promoting EJ by alt-EJ–MMEJ (Fig. 4*F*). In WT cells, Exo1 expression also decreased the level of Ku recovered at the DSB; however, the frequency of alt-EJ–MMEJ to NHEJ did not change nor did short-range resection (Fig. 4, *E* and *F*).

## Discussion

**Figure 5:**
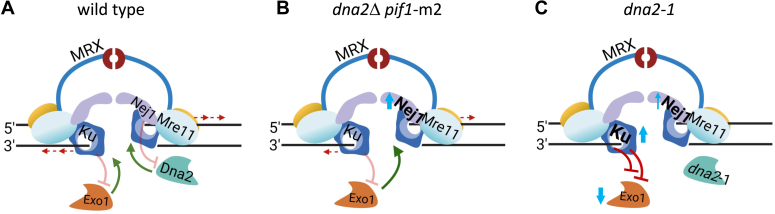
**Model depicting how dna2 mutants impact DSB repair, where the presence of nuclease-dead Dna2 at the break inhibits Exo1 nuclease through increased Ku.***A*, the schematic shows the antagonistic relationship between Ku70/80 (Ku) and Exo1 and Nej1 and Dna2. Ku binding to DNA ends at the break, inhibiting the access of Exo1 nuclease to perform 5′ resection. Nej1 is a competitive inhibitor of Dna2, blocking interactions between Dna2 and its binding partners at DSBs (1, 5, 6, 8, 9, 15, 16). *B*, upon deletion of *DNA2*, Nej1 increased, but Ku and Exo1 levels did not change. In *dna2*D *pif1*-m2 mutants, resection decreased approximately twofold as Exo1 was the only functional long-range nuclease at the DSB, and the frequency of alt-EJ–MMEJ markedly increased. *C*, in *dna2*-1 mutants, nuclease-dead Dna2 was recruited to the break site. Under this condition, there was a minor increase in Nej1. However, Ku increased, which in turn resulted in Exo1 inhibition. Therefore, in this mutant background, the functionality of both nucleases was compromised, resection was abrogated, and the frequency of NHEJ increased. The *dna2*-1 resection defect and phleomycin sensitivity were largely reversible either by ectopic expression of Exo1 or by deletion of Ku70. However, only Exo1 expression restored the balance of NHEJ and alt-EJ–MMEJ frequencies to levels observed in WT cells. alt-EJ, alt-end-joining; DSB, double-strand break; MMEJ, microhomology-mediated end joining; NHEJ, nonhomologous end-joining.

We set out to understand why *dna2*-1 nuclease-dead mutants were more sensitive to DSB-causing agents than *dna2*Δ mutants. Our results showed that Exo1 localization decreased in cells expressing nuclease-dead Dna2 and that overall resection in *dna2*-1 was reduced more than in cells where *DNA2* or *EXO1* were individually deleted. The negative effect of *dna2*-1 at DSB was caused by Ku-dependent inhibition of Exo1 localization (Fig. 5, *A* and *C*). The *dna2*-1 dominant-negative phenotype was largely overcome by either deleting *KU70* or expressing Exo1, as both rescued the resection defect and phleomycin sensitivity. By contrast, neither Exo1 recovery nor Ku70 recovery changed in *dna2*Δ *pif1*-m2; however, Nej1 levels markedly increased (Fig. 5*B*). Our work corroborates previous synthetic genetic array screening where *dna2*-1 in combination with *exo1*Δ was viable (30) and suggests that the SL resulting from deletion of both *DNA2* and *EXO1* stems from something other than a combined loss of nuclease activity, unless *dna2*-1 has activity *in vivo* below the level of detection. One possibility is that the *pif1*-m2 mutation, which is not present in *dna2*-1, might contribute to the genetic SL interaction as Pif1 and Exo1 were previously shown to coordinate checkpoint signaling at uncapped telomeres (22, 31).

The loss of Dna2 nuclease activity by a point mutation had a profound impact on the pathway used in DSB repair, and 5′ resection was central to the whole process. Ku and Nej1 are negative regulators of resection, and previous work by our laboratory and others showed there to be a division of labor for nuclease inhibition by these NHEJ factors (5, 6, 8, 9, 15, 16). Nej1 interacts with the binding partners of Dna2, including Mre11, Sae2, and Sgs1 to inhibit Dna2 activity. However, Nej1 does not inhibit Exo1 recruitment. By contrast, Ku binding at DSB directly inhibits the accessibility of Exo1 nuclease to DNA ends, and its recovery at the break site, whereas Dna2 can initiate resection in the presence of Ku (24, 25). Thus, the antagonistic relationship between Nej1 and Dna2 is distinct, and independent, from the antagonistic relationship between Ku and Exo1 at DSB. The data presented here highlight a layer of complexity not previously reported in earlier work, where Dna2 fidelity impacts the balance of Ku and Exo1 at the break, a relationship paramount for ensuring DSBs are repaired through the least mutagenic pathway available.

The *dna2* mutants impacted EJ differently in the reporter cell line where HR was precluded, a scenario relevant for G1 in the cell cycle. While NHEJ is error prone with the formation of small insertions and deletions, a more harmful outcome arises when resection initiates and more mutagenic alt-EJ–MMEJ proceeds. In *dna2*-1 mutants, the relative frequency of NHEJ increased and alt-EJ–MMEJ decreased, which was consistent with more Ku recovered at the break. The presence of Ku impacted the type of EJ repair in *dna2*-1 more than the resection defect. Both *dna2*-1 *exo1*Δ and *dna2*-1 *exo1*Δ ku70Δ mutants showed similar resection defects, but the increased frequency of NHEJ was Ku dependent (Fig. S3*C*). On the contrary, there was marked increase in alt-EJ–MMEJ in *dna2*Δ *pif1*-m2 mutants, which might have something to do with resection proceeding, albeit at reduced rate, in cells where *DNA2* was deleted. However, resection also equally decreased when *EXO1* was deleted, and repair occurred predominately through NHEJ (Figs. 3*B* and S3*C*). In all, these data might provide insight to the underlying cause of SL resulting from deletion of *EXO1* and *DNA2*, as HR and both EJ pathways would be disrupted.

The EJ observations also suggest that the presence of Ku was not the only regulatory factor determining the type of EJ repair. Rather, our data support a model wherein the frequency of NHEJ to alt-EJ–MMEJ was altered by the relative level of Ku in relation to other repair factors, namely Exo1 and Nej1. Exo1 promoted alt-EJ–MMEJ in the presence of Ku in *dna2* mutants, and we observed this under two experimental conditions. First, alt-EJ–MMEJ in *dna2*-1 mutants increased upon Exo1 expression (Fig. 4*E*), and second, alt-EJ–MMEJ increased in *dna2*Δ *pif1*-m2 mutants, where the level of Nej1 increased, but the levels of Exo1 and Ku70 were unaltered (Figs. 2, *A* and *B* and S3*C*). These phenotypic differences between *dna2*Δ and *dna2*-1 indicate the possibility of increased Nej1-dependent alt-EJ–MMEJ repair in *dna2*Δ cells (32, 33). Further work is needed to elucidate whether Nej1 has a role in regulating EJ; however, in support of this model, we recently reported that alt-EJ–MMEJ increased in aging cells as Ku declined, and Nej1 persisted DSBs (34).

Taken together, our data point out the dynamic interplay between Dna2 and Exo1 in DSB repair pathway choice and broaden the understanding of nuclease localization *versus* nuclease activity in DNA processing at break sites. The characterization of the two *dna2* mutants demonstrated that loss of Dna2 nuclease activity through different mutations resulted in different repair outcomes. The work has health relevance, and although *DNA2* deletion is embryonic lethal, point mutations are observed in diseases like Seckel syndrome and various kinds of cancer, with the corresponding P504→S mutation of *dna2*-1 in yeast seen in human cancers (35, 36).

## Experimental procedures

### Media details

All the yeast strains used in this study are listed in Table S1 with new ones obtained by crosses. The strains were grown on various media in experiments as described. For HO induction of a DSB, YPLG medium is used (1% yeast extract, 2% bacto peptone, 2% lactic acid, 3% glycerol, and 0.05% glucose). For the continuous DSB assay, YPA plates are used (1% yeast extract, 2% bacto peptone, and 0.0025% adenine) supplemented with either 2% glucose or 2% galactose. For the mating type assays, YPAD plates are used (1% yeast extract, 2% bacto peptone, 0.0025% adenine, and 2% dextrose).

### ChIP

ChIP assays were performed as previously described (5). Cells were cultured overnight in YPLG at 25 °C. Cells were then diluted to equal levels (5 × 10^6^ cells/ml) and cultured to one doubling (3–4 h) at 30 °C. 2% GAL was added to YPLG, and cells were harvested and crosslinked at various time points using 3.7% formaldehyde solution. Cut efficiencies for all strains are shown in Table S2. Following crosslinking, the cells were washed with ice-cold PBS, and the pellet was stored at −80 °C. The pellet was resuspended in lysis buffer (50 mM Hepes [pH 7.5], 1 mM EDTA, 80 mM NaCl, 1% Triton, 1 mM PMSF, and protease inhibitor cocktail), and cells were lysed using Zirconia beads and a bead beater. Chromatin fractionation was performed to enhance the chromatin-bound nuclear fraction by spinning the cell lysate at 13,200 rpm for 15 min and discarding the supernatant. The pellet was resuspended in lysis buffer and sonicated to yield DNA fragments (∼500 bp in length). The sonicated lysate was then incubated with αHA-, αFLAG-, or αMyc-antibody–conjugated beads or unconjugated beads (control) for 2 h at 4 °C. The beads were washed using wash buffer (100 mM Tris [pH 8], 250 mM LiCl, 150 mM [αHA and αFLAG] or 500 mM [αMyc] NaCl, 0.5% NP-40, 1 mM EDTA, 1 mM PMSF, and protease inhibitor cocktail), and protein–DNA complex was eluted by reverse crosslinking using 1% SDS in TE buffer, followed by proteinase K treatment and DNA isolation *via* phenol–chloroform–isoamyl alcohol extraction. qPCR was performed using the Applied Biosystem QuantStudio 6 Pro machine. PowerUp SYBR Green Master Mix was used to visualize enrichment at *MAT1* (0.15 kb from DSB), and *PRE1* was used as an internal control (Table S2). HO cutting was measured in strains used to perform ChIP in Table S3.

### Continuous DSB assay and identification of mutations in survivors

Cells were grown overnight in YPLG media at 25 °C to saturation. Cells were collected by centrifugation at 2500 rpm for 3 min, and pellets were washed 1× in ddH_2_O and resuspended in ddH_2_O. Cells were counted and spread on YPA plates supplemented with either 2% GLU or 2% GAL. About 1 × 10^3^ total cells were plated on glucose, and 1 × 10^5^ total cells were plated on galactose. The cells were incubated for 3 to 4 days at room temperature, and colonies were then counted on each plate. Survival was determined by normalizing the number of surviving colonies on the GAL plates to the number of colonies on the GLU plates. About 100 survivors from each strain were scored for the mating type assay as previously described (16), and at least 100 survivors were used to make a master plate, which was later replica-plated on -URA plates. The number of survivors on -URA plates was counted to determine the ratio of NHEJ and alt-EJ repair frequencies.

### qPCR-based ligation assay

As described previously (9), cells from each strain were grown overnight in 15 ml YPLG to reach an exponentially growing culture of 1 × 10^7^ cells/ml. Next, 2.5 ml of the cells were pelleted as “no break” sample, and 2% GAL was added to the remaining cells, to induce a DSB. About 2.5 ml of cells were pelleted after a 3 h incubation as time point 0 sample. After that, GAL was washed off, and the cells were released into YPAD, and respective time point samples were collected. Genomic DNA was purified using standard genomic preparation method by isopropanol precipitation and ethanol washing, and DNA was resuspended in 100 μl ddH_2_O. qPCR was performed using the Applied Biosystem QuantStudio 6 Flex machine. PowerUp SYBR Green Master Mix was used to quantify resection at HO6 (at DSB) locus. The *PRE1* locus was used as an internal gene control for normalization. Signals from the HO6/PRE1 time points were normalized to “no break” signals, and % Ligation was determined. The primer sequences are listed in Table S2.

### qPCR-based resection assay

Cells from each strain were grown overnight in 15 ml YPLG to reach an exponentially growing culture of 1 × 10^7^ cells/ml. Next, 2.5 ml of the cells were pelleted as time point 0 sample, and 2% GAL was added to the remaining cells, to induce a DSB. Following that, respective time point samples were collected. Genomic DNA was purified using standard genomic preparation method by isopropanol precipitation and ethanol washing, and DNA was resuspended in 100 ml ddH_2_O. Genomic DNA was treated with 0.005 μg/μl RNase A for 45 min at 37 °C. About 2 μl of DNA was added to tubes containing CutSmart buffer with or without inclusion of the *Rsa*I restriction enzyme and incubated at 37 °C for 2 h. qPCR was performed using the Applied Biosystem QuantStudio 6 Flex machine. PowerUp SYBR Green Master Mix was used to quantify resection at the *Rsa*I cut site 0.15 Kb DSB (in the *MAT1 locus*) and 4.8 Kb. *PRE1* was used as a negative control, and the primer sequences are listed in Table S2. *Rsa*I cut DNA was normalized to uncut DNA as previously described to quantify the %ssDNA (28). HO cutting was measured in strains for resection (Table S4).

## Data availability

All data are contained within the article, and all reagents are available upon request.

## Supporting information

This article contains supporting information (15).

## Conflict of interest

The authors declare that they have no conflict of interest with the contents of this article.
