# Supplementary material for: Loss of Dna2 fidelity results in decreased Exo1-mediated resection at DNA double-strand breaks
Source: J Biol Chem. 2024 Feb 2;300(3):105708. doi: 10.1016/j.jbc.2024.105708 (PMC10909748; doi:10.1016/j.jbc.2024.105708)
Supplement: Supporting Information [file mmc1.pdf]

Supporting Information

Loss of Dna2 nuclease activity results in decreased Exo1-mediated resection at DNA double strand breaks

Authors: Aditya Mojumdar, Courtney Granger, Martine Lunke and Jennifer A. Cobb

Figure S1. Sensitivity of various *dna2* and *sgs1* mutants to genotoxic stress

Figure S2. End-joining factors at DSBs in the dna2 mutants

Figure S3. HR factors at DSBs in *dna2* mutants

Figure S4. Resection in *pif1*-m2 mutants is unaltered by Exo1 expression

Table S1. *S. cerevisiae* strains used in this study

Table S2. Primers used in these studies

Table S3. DSB cut efficiency for strains used in ChIP

Table S4. DSB cut efficiency for strains used in resection assay

Supp Figure 1

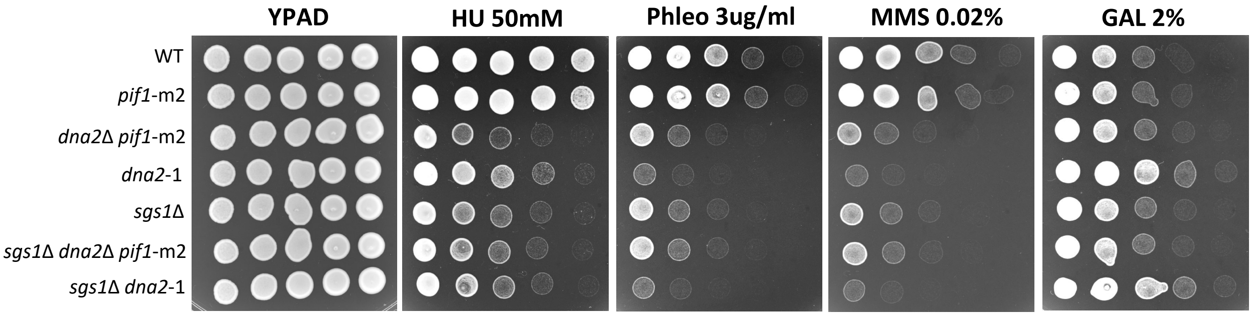

Figure S1. Sensitivity of various *dna2* and *sgs1* mutants to genotoxic stress

Five-fold serial dilutions of wild type (JC-727), *dna2*Δ *pif1*-m2 (JC-6005), *pif1*-m2 (JC-6006), *dna2*-1 (JC-6007), *sgs1*Δ (JC-3757), *sgs1*Δ *dna2*Δ *pif1*-m2 (JC-6101) and *sgs1*Δ *dna2*-1 (JC-5745) were spotted on YPAD, 50mM HU, 3.0 µg/ml phleomycin, 0.02% MMS and 2% GAL containing plates. These data are the same source images as in Figure 1A but shown here are the *sgs1*Δ mutant combinations also included in the experiment.

Supp Figure 2

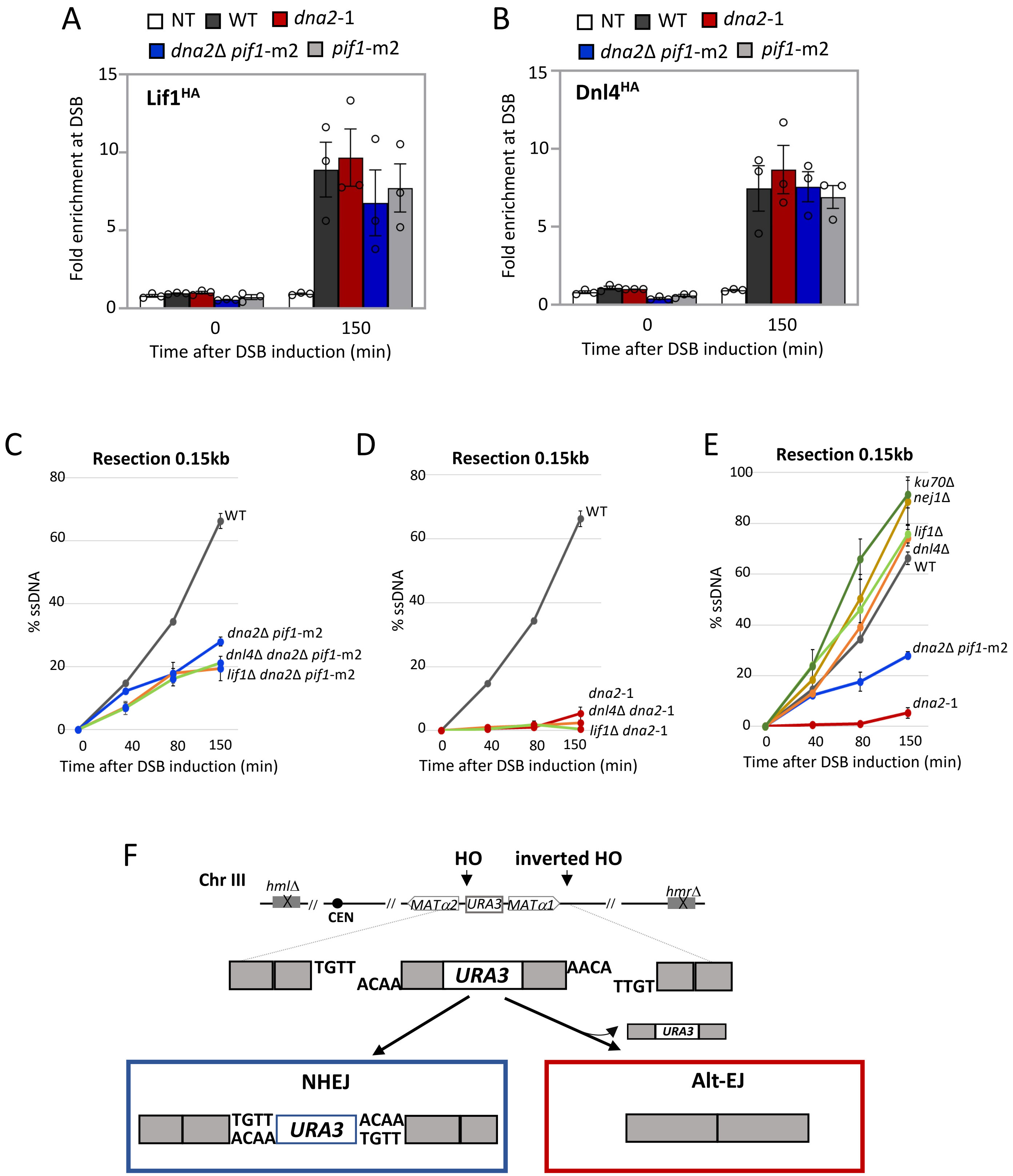

Figure S2. End-joining factors at DSBs in the dna2 mutants

(A) Enrichment of $Lif1^{HA}$ at 0.15kb from DSB, 0 min (no DSB induction) and 150 min after DSB induction in wild type (JC-3319), *dna2*-1 (JC-5834), *dna2*Δ *pif1*-m2 (JC-6110), *pif1*-m2 (JC-6136) and no tag control (JC-727).

(B) Enrichment of $Dnl4^{HA}$ at 0.15kb from DSB, 0 min (no DSB induction) and 150 min after DSB induction in wild type (JC-5672), *dna2*-1 (JC-5843), *dna2*Δ *pif1*-m2 (JC-6123), *pif1*-m2 (JC-6135) and no tag control (JC-727).

(C-E) qPCR based resection assay of DNA 0.15kb away from the HO DSB, as measured by % ssDNA, at 0, 40, 80 and 150 min post DSB induction in cycling cells in wild type (JC-727), *dna2*Δ *pif1*-m2 (JC-6005), *dna2*-1 (JC-6007), *ku70*Δ (JC-1904), *nej1*Δ (JC-1342), *lif1*Δ (JC-1343), *dnl4*Δ (JC-3290), *lif1*Δ *dna2*Δ *pif1*-m2 (JC-6121), *lif1*Δ *dna2*-1 (JC-5890), *dnl4*Δ *dna2*Δ *pif1*-m2 (JC-6119) and *dnl4*Δ *dna2*-1 (JC-5800).

(F) Schematic representation of regions around the two HO cut sites on chromosome III. Following HO endonuclease break induction, NHEJ repair joins the complementary ends of the *URA3* gene with the other ends of the break site. From two simultaneous cuts, noncomplementary ends are generated, in repair progresses through alt-EJ/MMEJ repair the *URA3* gene is deleted.

Supp Figure 3

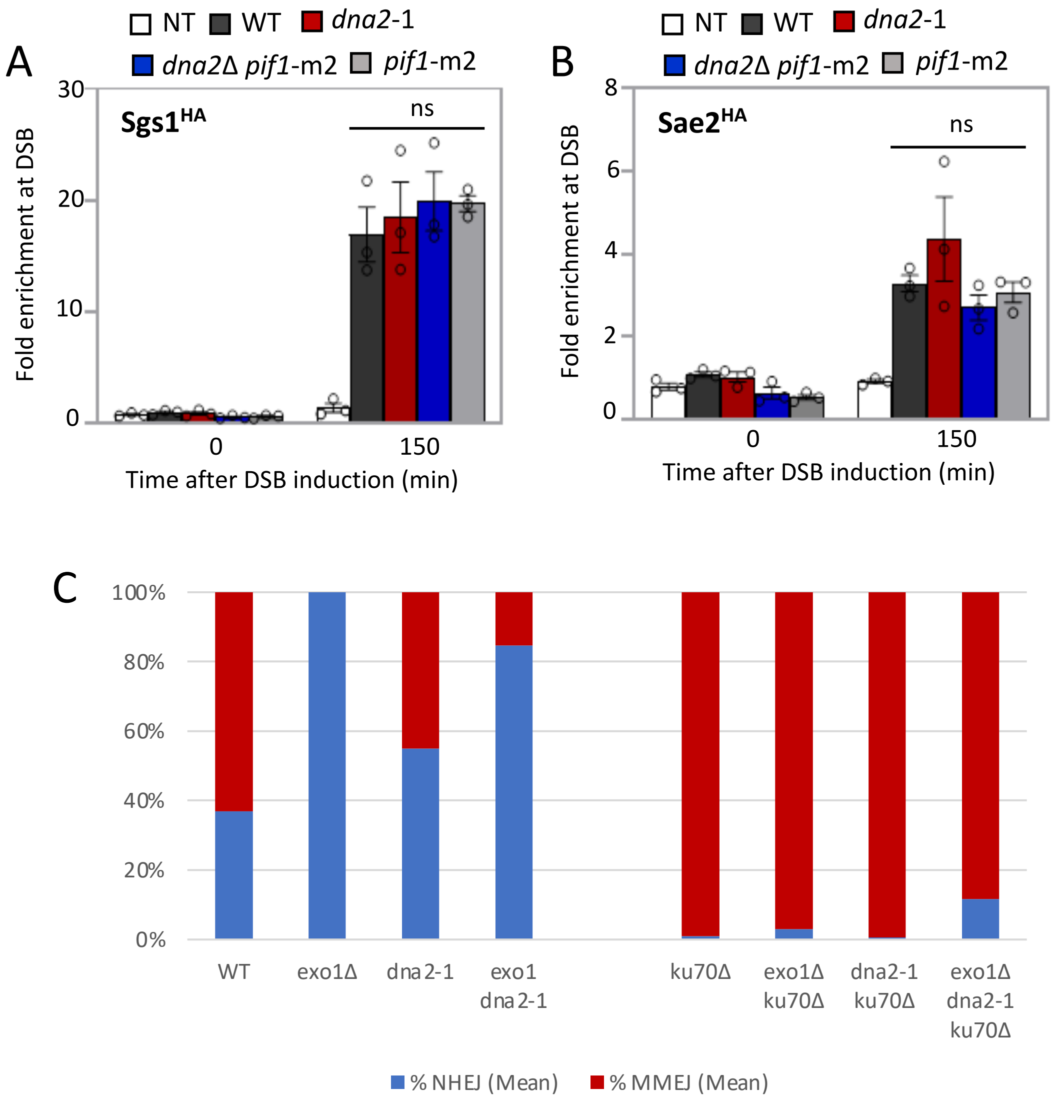

Figure S3. HR factors at DSBs in *dna2* mutants

(A) Enrichment of $Sgs1^{HA}$ at 0.15kb from DSB, 0 min (no DSB induction) and 150 min after DSB induction in wild type (JC-4135), *dna2*-1 (JC-5681), *dna2*Δ *pif1*-m2 (JC-6112), *pif1*-m2 (JC-6114) and no tag control (JC-727).

(B) Enrichment of $Sae2^{HA}$ at 0.15kb from DSB, 0 min (no DSB induction) and 150 min after DSB induction in wild type (JC-5116), *dna2*-1 (JC-5682), *dna2*Δ *pif1*-m2 (JC-6108), *pif1*-m2 (JC-6109) and no tag control (JC-727).

(C) Survival frequencies depicting the ratio of NHEJ (blue) and alt-EJ (red) repair frequencies in wild type (JC-5903), *exo1*Δ (JC-6222), *dna2*-1 (JC-6105), *exo1*Δ *dna2*-1 (JC-6328), *ku70*Δ (JC-6195), *ku70*Δ *exo1*Δ (JC-6272), *ku70*Δ *dna2*-1 (JC-6273) and *ku70*Δ *exo1*Δ *dna2*-1 (JC-6326).

Supp Figure 4

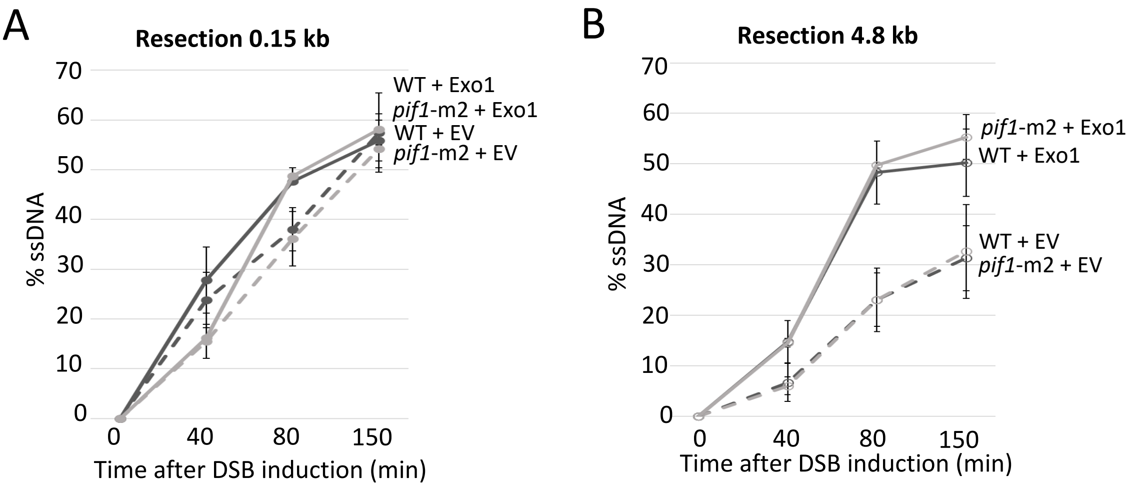

Figure S4. Resection in *pif1*-m2 mutants is unaltered by Exo1 expression

(A-B) qPC- based resection assay of DNA at two distances, 0.15kb and 4.8kb, from the HO DSB as measured by % ssDNA, at 0, 40, 80 and 150 mins. after DSB induction in cycling cells. Resection is compared in wild type (JC-727) *and pif1*-m2 (JC-6006) with 2-micron plasmid encoding Exo1 (pEM-EXO1) or empty vector (EV) [15].

Table S1: *S. cerevisiae* strains used in this study.

Strain	Genotype	Reference
JC-727	MAT α; *hml*::*ADE1 hmr*::*ADE1 ade3*::GAL-HO *ade1*-100 *leu*2-3, 112 *lys*5 *trp*1::*hisG ura3*-52	JKM179, [Lee et al. 1998]
JC-1342	JC-727 with *nej1*Δ::*KanMX6*	MAV015, [Valencia et al. 2001]
JC-1343	JC-727 with *lif1*Δ::*KanMX6*	Sorenson et al. 2017
JC-1687	JC-727 with *NEJ1-13MYC*::*TRP1*	Sorenson et al. 2017
JC-1904	JC-727 with *ku70*Δ::*KanMX6*	Sorenson et al. 2017
JC-3290	JC-727 with *dnl4*Δ::*KanMX6*	Sorenson et al. 2017
JC-3319	JC-727 with *LIF1-6HA*::*TRP1*	Sorenson et al. 2017
JC-3585	MAT a; *hml*::*ADE1 hmr*::*ADE1 ade3*::GAL-HO *ade1*-100 *leu*2-3, 112 *lys*5 *trp*1::*hisG ura3*-52	JKM179, [Lee et al. 1998]
JC-3757	JC-727 with *sgs1*Δ::*NatRMX4*	Sorenson et al. 2017
JC-3767	JC-727 with *exo1*Δ::*NatRMX4*	Sorenson et al. 2017
JC-3837	JC-3767 with *ku70*Δ::*KanMX6*	Sorenson et al. 2017
JC-3964	JC-727 with *KU70-FLAG*::*KanMX6*	This study
JC-4117	JC-727 with *DNA2-6HA::TRP1*	Mojumdar et al. 2019
JC-4135	JC-727 with *SGS1-6HA*::*TRP1*	Mojumdar et al. 2019
JC-4869	JC-727 with *EXO1-6HA::TRP1*	Mojumdar et al. 2022
JC-5116	JC-727 with *SAE2-6HA*::*TRP1*	Mojumdar et al. 2022
JC-5479	JC-1687 with *dna2-1*::*TS*	Mojumdar et al. 2022
JC-5670	JC-1342 with *dna2-1*::*TS*	Mojumdar et al. 2022
JC-5672	JC-727 with *DNL4-6HA::TRP1*	Mojumdar et al. 2022
JC-5681	JC-4135 with *dna2-1*::*TS*	This study
JC-5682	JC-5116 with *dna2-1*::*TS*	This study
JC-5692	JC-5655 with *exo1*Δ::*NatRMX4*	This study
JC-5707	JC-727 with *dna2-1-6HA::TRP1*	This study
JC-5800	JC-3290 with *dna2-1*::*TS*	This study
JC-5834	JC-3319 with *dna2-1*::*TS*	This study
JC-5843	JC-5672 with *dna2-1*::*TS*	This study
JC-5890	JC-1343 with *dna2-1*::*TS*	This study
JC-5903	MAT α; *ho MATalpha::*URA3::HOcs *hml::*ADE1 *hmr::*ADE1 *ade3::*GAL-HO	Ma et al. 2003
JC-5942	JC-1904 with *dna2-1*::*TS*	This study
JC-6005	JC-727 with *pif1-m2::KanMX6 dna2Δ::TS*	Mojumdar et al. 2022
JC-6006	JC-727 with *pif1-m2::KanMX6*	Mojumdar et al. 2022
JC-6007	JC-727 with *dna2-1*::*TS*	Mojumdar et al. 2022
JC-6018	JC-1904 with *EXO1-6HA::TRP1*	This study
JC-6020	JC-6007 with *EXO1-6HA::TRP1*	This study
JC-6025	JC-3837 with *dna2-1*::*TS*	This study
JC-6060	JC-6005 with *nej1*Δ::*KanMX6*	This study
JC-6062	JC-6006 with *nej1*Δ::*KanMX6*	This study

JC-6068	JC-6005 with *KU70-FLAG*::*KanMX6*	This study
JC-6069	JC-6006 with *KU70-FLAG*::*KanMX6*	This study
JC-6099	JC-6005 with *NEJ1-13MYC*::*TRP1*	This study
JC-6101	JC-6005 with *sgs1Δ*::*NatRMX4*	This study
JC-6103	JC-6006 with *sgs1Δ*::*NatRMX4*	This study
JC-6105	JC-5903 with *dna2-1*::*TS*	This study
JC-6108	JC-6005 with *SAE2-6HA*::*TRP1*	This study
JC-6109	JC-6006 with *SAE2-6HA*::*TRP1*	This study
JC-6110	JC-6005 with *LIF1-6HA::TRP1*	This study
JC-6112	JC-6005 with *SGS1-6HA*::*TRP1*	This study
JC-6114	JC-6006 with *SGS1-6HA*::*TRP1*	This study
JC-6115	JC-6005 with *EXO1-6HA::TRP1*	This study
JC-6117	JC-6006 with *EXO1-6HA::TRP1*	This study
JC-6119	JC-6005 with *dnl4Δ*::*KanMX6*	This study
JC-6121	JC-6005 with *lif1Δ*::*KanMX6*	This study
JC-6123	JC-6005 with *DNL4-6HA::TRP1*	This study
JC-6128	JC-6005 with *ku70Δ*::*KanMX6*	This study
JC-6129	JC-6006 with *ku70Δ*::*KanMX6*	This study
JC-6130	JC-6006 with *DNA2-6HA::TRP1*	This study
JC-6132	JC-6006 with *NEJ1-13MYC*::*TRP1*	This study
JC-6133	JC-6006 with *dnl4Δ*::*KanMX6*	This study
JC-6134	JC-6006 with *lif1Δ*::*KanMX6*	This study
JC-6135	JC-6006 with *DNL4-6HA::TRP1*	This study
JC-6136	JC-6006 with *LIF1-6HA::TRP1*	This study
JC-6181	JC-5903 with *pif1-m2::KanMX6 dna2Δ::TS*	This study
JC-6195	JC-5903 with *ku70Δ*::*KanMX6*	This study
JC-6213	JC-6128 with *EXO1-6HA::TRP1*	This study
JC-6215	JC-5942 with *EXO1-6HA::TRP1*	This study
JC-6222	JC-5903 with *exo1Δ*::*NatRMX4*	This study
JC-6237	JC-5655 with *KU70-FLAG*::*KanMX6*	This study
JC-6242	JC-3767 with *KU70-FLAG*::*KanMX6*	This study
JC-6272	JC-6195 with *exo1Δ*::*NatRMX4*	This study
JC-6273	JC-6105 with *ku70Δ*::*KanMX6*	This study
JC-6280	JC-6181 with *ku70Δ*::*KanMX6*	This study
JC-6326	JC-6273 with *exo1Δ*::*NatRMX4*	This study
JC-6328	JC-6105 with *exo1Δ*::*NatRMX4*	This study

Table S2: Primers used in these studies.

Primer Name	Primer Sequence (5'-3')
HO6 Forward Primer	AATATGGGACTACTTCGCGCAACA
HO6 Reverse Primer	CGTCACCACGTACTTCAGCATAA
MAT1(.15 kb RsaI) Forward Primer	CCTGGTTTTGGTTTTGTAGAGTGG
MAT1 (.15 kb RsaI) Reverse Primer	GAGCAAGACGATGGGGAGTTTC
PRE1 Forward Primer	CCCACAAGTCCTCTGATTTACATTCG
PRE1 Reverse Primer	ATTCGATTGACAGGTGCTCCCTTTTC
4.8 kb RsaI Forward primer	CTCATCTGTGATTTGTGGAT
4.8 kb RsaI Reverse primer	GAGGCAAGTAGATAAGGGTA

Table S3: DSB cut efficiency for strains used in ChIP.

Strain	DSB cut % at 150min
JC-3964	95.65
JC-4117	98.94
JC-4869	99.27
JC-5707	98.93
JC-6018	98.36
JC-6020	98.82
JC-6068	92.63
JC-6069	84.81
JC-6115	84.58
JC-6117	80.90
JC-6130	95.65
JC-6215	98.94
JC-6237	99.27
JC-6242	98.93

Table S4: DSB cut efficiency for strains used in resection assay.

Strain	DSB cut (%)		
	40min	80min	150min
JC-727	50.95	85.95	95.45
JC-1904	89.11	88.98	97.66
JC-3757	52.12	85.68	94.85
JC-3767	51.67	77.16	91.32
JC-3837	91.96	97.71	99.04
JC-5372	75.22	88.84	93.10
JC-5655	39.69	79.06	90.56
JC-5669	33.13	79.71	83.59
JC-5673	51.12	69.31	81.26
JC-5692	50.54	65.28	81.45
JC-5745	76.57	92.11	97.03
JC-5748	46.41	79.62	91.65
JC-5942	91.68	98.00	98.96
JC-6005	94.39	99.13	99.09
JC-6006	82.96	93.15	94.72
JC-6025	78.37	93.97	94.31
JC-6098	75.89	94.17	95.25
JC-6101	76.68	84.78	94.48
JC-6128	77.22	83.92	93.96
JC-6152	84.61	89.88	90.49
